# COVID-19 vaccine hesitancy: a systematic review of barriers to the uptake of COVID-19 vaccine among adults in Nigeria

**DOI:** 10.1186/s42269-023-01017-w

**Published:** 2023-03-21

**Authors:** Tolulope Babatope, Vera Ilyenkova, Debbi Marais

**Affiliations:** 1 University of Essex Online: Kaplan Open Learning Essex Ltd, London, UK; 2 Warwick Medical School, University of Warwick, Gibbet Hill Rd, Coventry, UK

**Keywords:** Adults, COVID-19, COVID-19 vaccines, Coronavirus, Nigeria, Vaccine hesitancy

## Abstract

**Background:**

Since the outbreak of coronavirus (COVID-19) disease was reported in 2019, huge human and material resources have been expended globally to combat the spread of the disease. Achieving herd immunity through mass vaccination remains an important strategy to adopt in the war against this disease since it is practically impossible for 60–70% of the population to achieve immunity through natural infection alone. Unfortunately, there have been widespread reports of COVID-19 vaccine hesitancy. This study aims to systematically review the literature to provide an up-to-date assessment of COVID-19 vaccine acceptance rates and also explore factors impacting COVID-19 vaccine hesitancy among adults in Nigeria.

**Main body of the abstract:**

A systematic search of indexed electronic peer-reviewed literature published from 2019 onwards was conducted in Science Direct, PubMed, ProQuest, and EBSCOhost databases and reported according to the PRISMA checklist and Synthesis without meta-analysis (SWiM) in systematic review reporting guidelines. Fifteen out of the 148 studies retrieved, met the inclusion criteria and these were critically appraised using the Centre for Evidence-Based Medicine Critical Appraisal checklist and Mixed Methods Appraisal Tool, version 2018. Basic descriptive statistic (percentage) was employed in the analysis of acceptance rates of the COVID-19 vaccine among various subgroups of adults in Nigeria, while a thematic analysis of the facilitators and barriers to the uptake of the COVID-19 vaccine in Nigeria was conducted. Acceptance rates ranging from 24.3% to 49.5% were observed across the four studies conducted among the high-risk populations in Nigeria, while the acceptance rates among the low-risk populations ranged from 26.0% to 86.2%. Themes such as socio-demographic factors, perception of risk factors, and concerns about the vaccine's safety and efficacy act interchangeably as facilitators and barriers to the uptake of COVID-19 vaccines, whereas political factors, conspiracy theories, and cost primarily act as barriers to vaccine uptake.

**Short conclusion:**

Substantial heterogeneity was observed in COVID-19 vaccine acceptance rates among adults in Nigeria. More than half of the studies reviewed reported acceptance rates below 60.0%. A multidisciplinary approach is recommended in engaging important stakeholders, to effectively address COVID-19 vaccine hesitancy in Nigeria.

## Background

A novel communicable disease, the COVID-19 infection, was first reported in Wuhan, Hubei province in China on November 17, 2019 (Bryner 2020). After a rapid spread to every continent of the world, the World Health Organization (WHO) classified the outbreak as a pandemic in March 2020. COVID-19 is a communicable respiratory disease caused by a virus belonging to the coronavirus family and known as the severe acute respiratory syndrome coronavirus 2 (SARSCoV2). As of mid-January 2022, a total of 335 million cases of COVID-19 infections had been recorded worldwide and 5.5 million deaths occurred as a result of this deadly disease (Worldometer 2022). Furthermore, over 10,000 frontline healthcare workers were infected across Africa (WHO 2021).

Although vaccines generally require years of research and testing before they can be used in clinical settings, the COVID-19 pandemic led scientists into a race to generate safe and effective coronavirus vaccinations in record time in 2020. As of June 13, 2022, about 122 COVID-19 vaccines were already undergoing human clinical trials, and 49 were nearing completion while 12 vaccines had been approved for human use (Corum et al. 2022). Despite the approval of some of these COVID-19 vaccines, for the prevention of the COVID-19 disease, (BNT162b vaccine produced by Pfizer, ChAdOx1 nCoV-19 vaccine produced by Oxford–AstraZeneca, Moderna, Sputnik V, and Johnson & Johnson) the uptake of these vaccines has been suboptimal worldwide (Sallam et al. 2021).

The term "vaccine hesitancy" refers to the reluctance of some individuals within the population to receive safe and recommended available vaccines. With regards to its scope, vaccine hesitancy can be conceptualised as existing on a continuum that extends from individuals who accept all vaccines without any reservations to individuals who refuse all vaccines without any reservations, with individuals who are vaccine-hesitant making up the diverse groups that exist between these two extremes (MacDonald 2015). Vaccine hesitancy has been widely researched and documented as a phenomenon (Dubé et al. 2018). This concept existed before the COVID-19 crisis and over the years has negatively impacted the efforts being made to control and eradicate some infectious diseases such as poliomyelitis (Machingaidze and Wiysonge 2021). In addition, vaccine hesitancy has been designated as one of the top ten obstacles to attaining global health by the WHO, a phenomenon capable of reversing the advancements made in combating vaccine-preventable diseases (WHO 2019).

COVID-19 vaccine hesitancy has been documented by researchers in high, middle, and low-income countries all over the world (Dhama et al. 2021; Sallam 2021; Aw et al. 2021). This situation is not specific to any country, community, or religion. As early as the summer of 2020, researchers and professionals in the field of public health voiced their concerns regarding the existence of COVID-19 vaccine hesitancy in the United States of America (Khubchandani et al. 2021). Anti-vaccine campaigners were spreading rumours that coronavirus vaccinations were being used to implant microchips. A now-deleted YouTube video made in 2020 pushing pandemic conspiracy theories and claiming immunisation would "kill millions" had over 8 million views (Ball 2020).

## Main text

### COVID-19 vaccine hesitancy in Nigeria

Nigeria was the first country in Sub-Saharan Africa to announce a confirmed case of COVID-19 infection, and this index case was reported on February 27, 2020 (Siwatu et al. 2020). As of May 27, 2022, Nigeria had recorded a total of 256,028 confirmed cases of COVID-19 across all states and 3,143 deaths (NCDC 2021). The pandemic disproportionately affected males with a male/female ratio of 60:40 cases and 74:26 deaths. Individuals over the age of 50 accounted for 70% of the mortality burden. All thirty-six states are affected, but Lagos and the Federal Capital Territory reported the most cases (NCDC 2021). However, it's possible that these numbers don't accurately reflect the severity of COVID-19 disease in Nigeria because the country had only tested 5,160,280 people at the time of this report, despite a population of around 200 million (NCDC 2021). Also, in the year 2020, some people died in Kano State, and the cause of these deaths remain unknown (Akinwotu and Burke 2020).

In compliance with the global trend, the Nigerian government instituted public health measures by prohibiting public gatherings and restricting movement and businesses to combat the infection. However, compliance with these public health measures was a great challenge in a country such as Nigeria where the relationship between the government and the populace is marred by a lack of trust. Other factors such as poverty, overcrowding and lack of potable water also made compliance with these public health measures difficult in many sub-Saharan African countries including Nigeria (Solomon et al. 2022).

Although the incidence of COVID-19 infection has been on a downward trend suggesting a decrease in cases, the second wave experienced by the country in December 2020, and non-adherence to non-pharmaceutical preventive measures underscores the need for a vaccine. It is important to note that throughout its history, Nigeria has struggled with vaccine hesitancy and refusal, which over the years resulted in the loss of public confidence in vaccination programmes. For instance, the polio vaccine boycott in Northern Nigeria in 2003 was sparked by rumours that the polio vaccine contained anti-fertility chemicals and HIV (Yahya 2007). Following the vaccine boycott, wild polio re-infection was experienced in 20 African and Asian countries. The Nigerian government engaged numerous stakeholders, such as religious leaders, traditional rulers, non-governmental organisations, vaccine programme officers, and World Health Organisation representatives, in order to restore public trust and end the oral polio vaccine boycott.

At first, the inadequate supply of COVID-19 vaccine in Nigeria was the primary reason for the country's low vaccination rate, since the vaccines were manufactured outside of Nigeria. However, with an increase in supply, the supply-and-demand dynamic took effect, and vaccine acceptance became a crucial factor in determining coverage. Low COVID-19 uptake has significant negative consequences, which cannot be overemphasised. For instance, achieving herd immunity to disrupt the spread of this infection is nearly impossible without a high vaccination rate. For herd immunity to be achieved based on current estimates, more than 60% of the population would need to have either a natural COVID-19 infection or vaccination (Altmann et al. 2020).

**Fig. 1:**
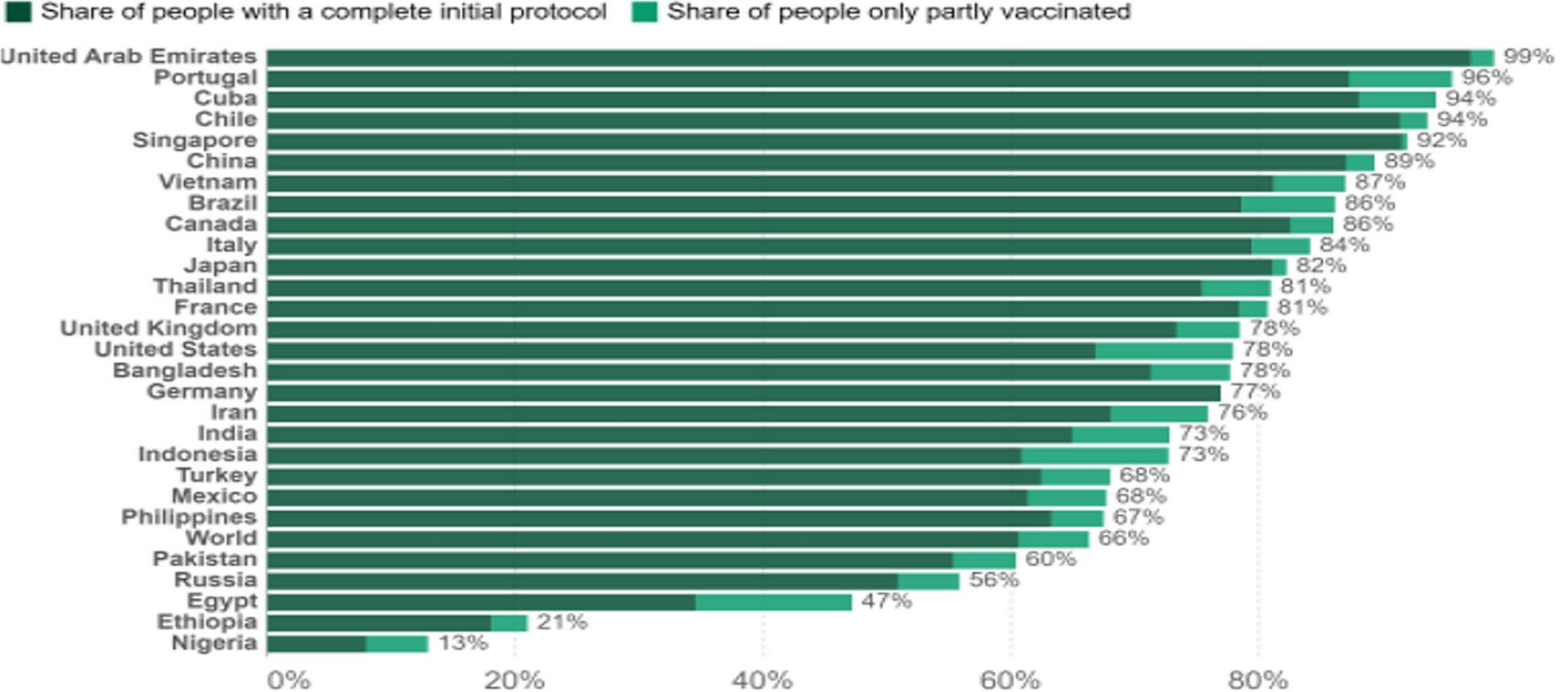
Share of people vaccinated against COVID-19 globally, June 18, 2022

Nigeria and the majority of African nations did not meet the December 2021 vaccination target of 40 per cent. According to the data obtained from the National Primary Health Care Development Agency (NPHCDA), the number of fully vaccinated individuals was 9.8 per cent (10,925,624) as of March 19, 2022, and health experts are concerned that the country will not meet the 70 per cent target by June 2022 if drastic action is not taken (Obinna 2022). Similarly, global data indicated that only 13% of the Nigerian population had been fully vaccinated as of June 18, 2022 as depicted in Fig. 1 below (Ritchie et al. 2022).

Furthermore, studies conducted in Northern Nigeria reported that acceptance of the COVID-19 vaccine was less than optimal among adults in the metropolitan area of Kano (Iliyasu et al. 2021a). Also, a cross-sectional survey conducted at a Nigerian university in the eastern part of Nigeria reported a COVID-19 vaccine hesitancy rate of 65.04% (Uzochukwu et al. 2021). This high hesitancy rate is disconcerting for a tertiary institution and warrants exploration into the root causes of COVID-19 vaccine hesitancy in Nigeria.

It is imperative to end the pandemic because it has significant negative impacts on the economy, mental health, education, and social activities globally. The International Monetary Fund predicts an output loss of close to $9 trillion for the global economy due to this pandemic over the next 2 years (IMF 2020). Therefore, it is critical to understand the various reasons why Nigerians accept or reject the COVID-19 vaccine and the dynamics of COVID-19 vaccine hesitancy in Nigeria. This will allow the government and other policymakers to tailor health promotion interventions to address the specific causes of COVID-19 vaccine hesitancy among the Nigerian population.

This study therefore aims to systematically review the literature to explore factors impacting COVID-19 vaccine hesitancy among adults in Nigeria while the objectives of the study are as follow (i) To compare acceptance rates of the COVID-19 vaccine among high- and low-risk adults in Nigeria (ii) To explore the perceptions of adults in Nigeria regarding facilitators and barriers to COVID-19 vaccine acceptance.

### Methods

#### Study design

This systematic review was guided by the Synthesis without meta-analysis (SWiM) in systematic review reporting guidelines and the PRISMA checklist (Moher et al. 2016). The research protocol was registered on PROSPERO (Prospero registration number: CRD42022314382) after an initial search on Google scholar and PROSPERO showed that no systematic review on the facilitators and barriers to the uptake of COVID-19 vaccine among various subgroups of adults in Nigeria had been conducted.

#### Search strategy

A systematic search of indexed peer-reviewed literature published from 2019 onwards was conducted in the following electronic databases: Science Direct, PubMed, ProQuest, and EBSCOhost (Medline and Cinahl). To ensure retrieval of relevant articles, database searches were carried out using keywords, phrase searching and controlled vocabulary searching through database-specific indexing terms. Boolean operators, truncation and field tags were also incorporated. The search terms containing relevant keywords were combined as follows “COVID-19 Vaccines" **OR** "Coronavirus Vaccin*" **OR** "SARS COV 2 Immunisation" **OR** "Covid 19 Vaccin*" **AND** Hesitancy **OR** Decline **OR** Refusal **AND** Acceptance **OR** Uptake **AND** Nigeria **OR** Nigerian **OR** Nigerians.

#### Screening and eligibility

Only studies that met the following inclusion criteria were included: (1) Research publications focusing on all adult populations (> 18 years) eligible for COVID-19 vaccination in Nigeria for instance high-risk populations e.g. healthcare workers, adults with underlying comorbidities or underlying illnesses and low-risk populations e.g. students in tertiary institutions, other adults who are not frontline workers (2) Observational (quantitative, qualitative and mixed-method) studies evaluating or reporting primary and secondary data on factors impacting COVID-19 vaccine uptake in Nigeria (3) Studies published from 2019 onward (4) Studies published in peer-reviewed journals in the English language. According to the American Centre for Disease Control, high-risk individuals are those with an increased risk of developing severe illness if infected with the COVID-19 virus and these include frontline healthcare workers, individuals with underlying chronic medical conditions such as hypertension, diabetes, chronic renal failure, bronchial asthma, cancer, and immunosuppressive states e.g. HIV/AIDS (CDC 2022). Excluded articles include: (1) Studies evaluating barriers to the uptake of other vaccines in Nigeria e.g. HPV or POLIO vaccines (2) Studies evaluating barriers to the uptake of other COVID-19 prevention methods e.g. Physical distancing, isolation and face masks (3) Literature reviews (4) Editorials and opinion pieces and (5) Book chapters.

Rayyan, an online reference management software, was utilised to organise peer-reviewed articles and manage the screening process. After duplicates were removed, the remaining articles were screened in phases to decrease the number of texts. The first stage involved title and abstract screening. Two reviewers (TB and VI) independently performed the screening, while a third reviewer (DM) resolved conflicts. This was followed by the second stage of double independent screening in which full-text screening of articles was conducted.

#### Critical appraisal

The eligible articles were critically appraised for methodological quality using the Centre for Evidence-Based Medicine Critical Appraisal checklist for cross-sectional studies, while the Mixed Methods Appraisal Tool (MMAT), version 2018 was used for the critical appraisal of quantitative descriptive and mixed method studies. Studies were classified into good, fair, and poor quality based on the level of compliance with the criteria in the assessment tools.

#### Data extraction and analysis

Data extraction from the included peer-reviewed studies was conducted using Microsoft Excel. Demographic information and key data extracted from each study include citation (the author's name and the year of the study), country of study, the aim of the study, study population characteristics, study setting, study design, sample size, sampling technique, sample analysis method and data source. The level of acceptability of COVID-19 vaccines among high-risk and low-risk adults in Nigeria was analysed, summarised and compared using basic descriptive statistics (percentages), while a thematic analysis of the facilitators and barriers to the uptake of COVID-19 vaccine was conducted using the inductive coding approach.

#### Ethical consideration

This systematic review is an evaluation of published, publicly accessible studies and did not require the collection of original data or any interaction with human subjects. Ethical approval was obtained from the University of Essex Online, United Kingdom.

## Results

**Fig. 2:**
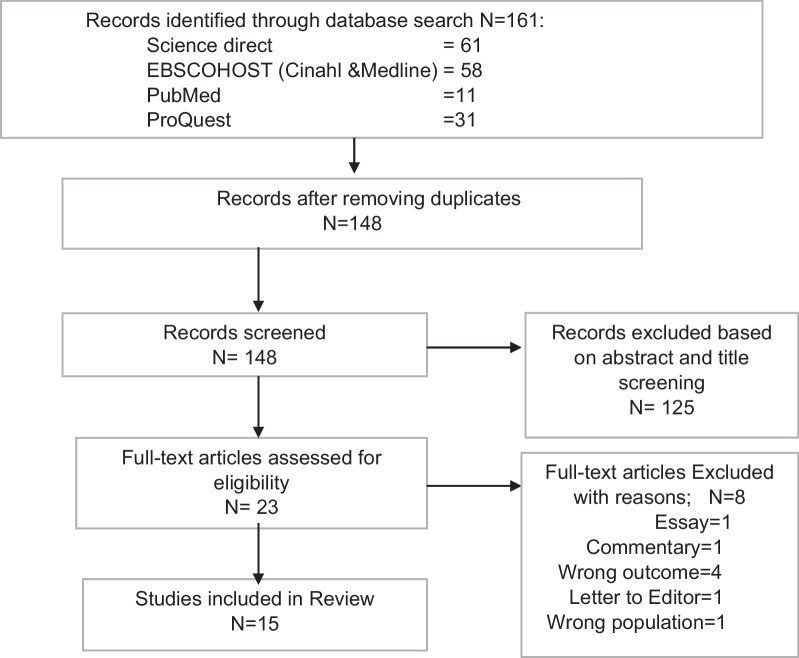
PRISMA flow chart

A total of 161 records identified from the four databases were exported into Rayyan systematic review software and duplicates were removed, leaving 148 records to be screened (Fig. 2). The kappa test of inter-rater agreement between reviewers was 0.83. Full-text screening was conducted to further assess the remaining 23 articles for eligibility before data extraction. Eight articles were excluded mainly due to being the wrong publication type (*n* = 4) or having the wrong outcome (*n* = 4). At the end of the process, 15 articles were eligible for inclusion in the review with an inter-rater agreement of κ = 0.95.

### Study characteristics

The main characteristics of studies included in the review are summarised in Table 1. Tools used by researchers to assess COVID-19 vaccine acceptance and hesitancy were pretested self-administered paper-based and online questionnaires (Google forms). For the qualitative part of the mixed-method studies, in-depth interviews were conducted by researchers with the recordings transcribed word-for-word, and a thematic analysis was conducted using the "Framework Approach”.

**Table 1:** Summary table of study characteristics

S/N	Citation	Country of Study	Aim of the study	Study population Characteristic	Study Setting	Study design	Sample Size	Sample technique	Analysis	Data source
1	Iliyasu et al. (2021b)	Nigeria	To assess the predictors of acceptability of Covid-19 vaccines and reasons for vaccine hesitancy among members of staff of a tertiary hospital in Kano State, Northern Nigeria	Clinical and non-clinical staff of the hospital	Hospital-based	Sequential explanatory mixed method study (Structural survey and in-depth interview)	343	Stratified Purposive	SPSS version 22 Thematic analysis using the framework approach	Primary data from survey respondents and in-depth interview participants
2	Adebisi et al (2021)	Nigeria	To understand the perception of social media users regarding a hypothetical COVID-19 vaccine in Nigeria	Adult male and female social media users in Nigeria	Web-based	Cross-sectional survey	517	Non-probability convenient sampling	STATA 14 software Simple descriptive analysis Inferential Statistics (Chi-square test)	Primary data from survey
3	Iliyasu et al. (2021a)	Nigeria	1. To assess predictors of acceptability of COVID-19 vaccine among adults in urban Kano 2. To identify reasons for vaccine hesitancy among adults in urban Kano Northern Nigeria	Adults in Tarauni and Nassarawa Local government areas of metropolitan Kano	Community-based	Mixed method design	450	Multi-stage sampling method	SPSS version 22 Logistic regression Thematic analysis using the framework approach	Primary data from survey respondents and in-depth interview participants
4	Agha et al (2021)	Nigeria	To understand drivers of COVID-19 vaccination uptake among healthcare workers in Nigeria	Adults Facebook users in Nigeria	Web-based	Cross-sectional survey	496	Stratified sampling	Basic descriptive statistics Multivariate analysis	Primary data from survey respondents
5	Kayanda et al., (2021)	Sub-Saharan African countries	To estimate willingness to accept the COVID-19 vaccine and identify differences in acceptance across countries and population groups	Adults’ participants in High- frequency phone surveys	Telephone-based	Quantitative descriptive	416 million people (38% of the population of Sub-Saharan Africa)	Random sampling	Multivariate logistic regression Descriptive statistics	Cross-country comparable data from 6 sub-Saharan African countries
6	Okafor et al (2021)	Nigeria	To assess the acceptability and willingness to pay for hypothetical COVID-19 vaccine among Nigerians	Adults with internet access	Web-based	Cross-sectional survey	770	Not stated	Multivariate logistic regression SPSS version 25	Primary data from phone survey respondents
7	Solis Arce et al. (2021)	Asia Africa South America Russia	To analyse COVID-19 acceptance across 15 survey samples covering 10 low and middle-income countries	Adults with access to mobile phones	Telephone-based	Cross-sectional survey	44,260	Random sampling Random digit dialling	R-software version 4.0.4 Random effect meta-analysis model	Primary data from phone survey respondents
8	Amuzie et al (2021)	Nigeria	To assess the socio-demographic factors associated with COVID-19 vaccine hesitancy among healthcare workers in Abia State, southeast Nigeria	Healthcare workers	Hospital-based	Cross-sectional survey	416	Simple random sampling	SPSS version 26 Bivariate analysis Multiple logistic regression Descriptive Statistics	Primary data from phone survey respondents
9	Uzochukwu et al (2021)	Nigeria	To estimate the proportion of the Nnamdi Azikwe University community willing to be vaccinated against COVID-19, level of hesitancy and its associated factors	Adult students and staff of the University	Community-based	Cross-sectional surveys	349	Convenience sampling technique	SPSS version 23 and Minitab version 19 Inferential statistics- Chi-square test Descriptive statistics	Primary data from phone survey respondents
10	Adigwe (2021)	Nigeria	To investigate the factors associated with vaccine hesitancy and willingness to pay for COVID-19 vaccination	Adults residing in Abuja metropolis	Community and web-based	Cross-sectional surveys	1767	Snowball sampling strategy	SPSS version 25 Inferential statistics- Chi-square test Descriptive statistics	Primary data from phone survey respondents
11	Eze et al (2021)	Nigeria	To assess the determinants of the COVID-19 vaccine acceptability among Nigerians	Adults in schools Corporate organisations, residential and, recreational areas and faith-based institutions	Population -based	Cross-sectional study	360	Simple random sampling	SPSS version 22, Inferential statistics, Chi-square test. Bivariate analysis	Primary data from phone survey respondents
12	Iliyasu et al (2022)	Nigeria	To assess the acceptability of the COVID-19 vaccine and identify predictors of vaccine hesitancy among people living with HIV/AIDS (PLWHIV) in a tertiary hospital in Kano State, Nigeria	Adult patients living with HIV	Clinic-based	Sequential explanatory mixed-method study	360	Systematic sampling	SPSS version 22 Binary Logistic regression Thematic analysis using the framework approach	Primary data from survey respondents and in-depth interview participants
13	Adedeji- Adenola et al., (2022)	Nigeria	To assess the factors influencing the awareness, perception and willingness to receive the COVID-19 vaccine among Nigerian adults	Adults-male and female, social media users in Nigeria	Web-based	Cross-sectional survey	664	Snowball sampling technique	SPSS version 24 and Microsoft Excel Logistic regression model	Primary data from survey respondents
14	Harapan et al., (2022)	Asia Africa South America	To determine the level of COVID-19 vaccine hesitancy among communities	Adults in Asia, Africa, and South America with internet access	Web-based	Cross -sectional study	1832	Not stated	SPSS software Logistic regression model	Primary data from survey respondents
15	Anjorin et al (2021)	Africa	To assess the potential for COVID-19 vaccine hesitancy and its determinants among Africans	Adult social media users	Web-based	Cross-sectional Continent-wide (Africa) survey	5416	Convenience Sampling method	Descriptive Statistics Chi-Square test Bivariate logistic regressive model	Primary data from survey respondents

### Key observations

#### Acceptance rates of COVID-19 vaccines among high-risk populations in Nigeria

Four out of the 15 articles included in this systematic review assessed the acceptance rates of the COVID-19 vaccine among high-risk populations.

**Fig. 3:**
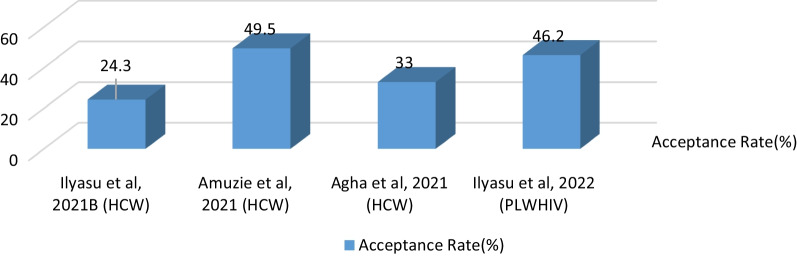
Acceptance rates of COVID-19 vaccine among high-risk populations in Nigeria (%)

A range of acceptance rates from 24.3 to 49.5% was observed across the four studies conducted among the high-risk populations in Nigeria (Fig. 3). In addition, the study conducted by Agha et al. (2021) showed that only 33.0% of the respondents had taken two doses of the COVID-19 vaccine as of July 2021.

#### Acceptance rates of COVID-19 vaccine among low-risk populations in Nigeria

**Fig. 4:**
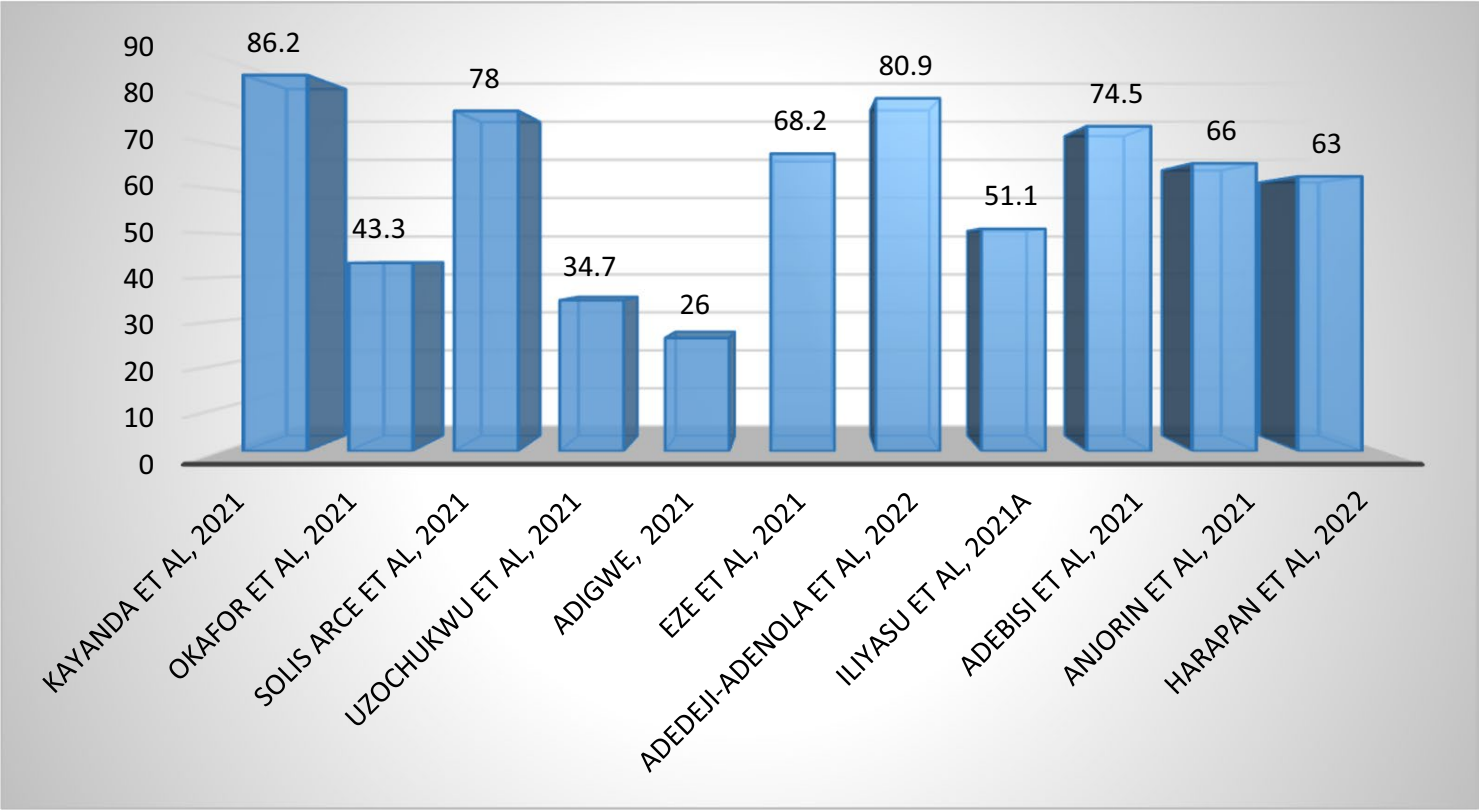
Acceptance rate of COVID-19 vaccine among low-risk adults in Nigeria (%)

Online and telephone surveys on willingness to accept the COVID-19 vaccine in African countries, including Nigeria, reported acceptability rates ranging from 26.0% to 86.2% (Fig. 4) (Solís Arce et al. 2021; Anjorin et al. 2021; Harapan et al. 2022). Furthermore, acceptability rates of 66.2%, 51.1%, 34.7%, 43.3% and 74.5%, respectively, were reported among adult populations in various Nigerian states. In addition, only 26.0% of the adult populations surveyed in the Federal Capital Territory (FCT) were willing to accept and pay for the COVID-19 vaccine (Adigwe 2021).

#### Facilitators of COVID-19 vaccine uptake among adults in Nigeria

The analysis of data using the inductive coding approach produced six themes (categories) with regard to the facilitators and barriers to the uptake of the COVID-19 vaccine among adults in Nigeria.

Key factors responsible for the acceptability of the COVID-19 vaccine include socio-demographic factors, perception of risk factors, and concerns about the safety and efficacy of the vaccine. As shown in Table 2, older age group, high socioeconomic status, higher level of education, male gender, and living in urban areas are the socio-demographic factors that act as facilitators of COVID-19 vaccine uptake in the majority of the studies examined (Iliyasu et al. 2021a; Agha et al. 2021; Adigwe 2021; Adedeji-Adenola et al. 2022; Amuzie et al. 2021; Eze et al. 2021). However, discrepancies were observed in two web-based studies conducted among social media users in Nigeria to assess the acceptability and willingness to pay for hypothetical COVID-19 vaccines (Adebisi et al.^2021^; Okafor et al. 2021). In these studies, younger adults were statistically significantly associated with high COVID-19 uptake.

**Table 2:** Facilitators of COVID-19 vaccine acceptance among adults in Nigeria

S/N	Sociodemographic factors	Perception of risk factors	Concerns about COVID-19 vaccine safety and efficacy
1. Iliyasu et al., (2021b)	Older age group High-income earners/ High socioeconomic status	Presence of chronic medical conditions Being a healthcare provider (Physicians, nurses)/ having greater than 10 years of work experience as a healthcare worker	The belief that COVID-19 vaccines are safe is associated with high vaccine acceptability
2.Adebisi et al (2021)	Younger adults associated with willingness to take **hypothetical** COVID-19 vaccine Being a respondent from the southern part of Nigeria associated with higher vaccine uptake		
3.Iliyasu et al., (2021a)	Older age group High socioeconomic status High acceptability observed among respondents in South-south and Southwestern parts of Nigeria	Chronic medical disorders are statistically significantly associated with vaccine acceptability	High vaccine acceptability was observed among those indifferent to infertility related rumours
4. Agha et al (2021)	A higher level of vaccination was observed among individuals with higher level of education (Bachelor's or Master's degree)	Being a Physician associated with taking two doses of COVID-19 vaccination	
5. Kayanda et al., (2021)	Acceptance of the COVID-19 vaccine is higher among the male gender, those with lower levels of education and poorer households in the Nigerian sample		
6. Okafor et al (2021)	Younger people were more willing to receive **hypothetical** COVID-19 vaccine. Muslim respondents associated with vaccine acceptance	Having a COVID-19 test done previously was associated with higher vaccine uptake	
7. Solis-Arce et al., (2021)	Respondents aged 25–54 years old were more willing to take the COVID-19 vaccine in Nigerian sample		
8. Amuzie et al (2021)	Older age group High-income earners Marital status (widowed, divorced or married) associated with higher level of COVID-19 vaccine acceptability	Clinical staff like Doctors, nurses and allied healthcare professionals had a higher level of COVID-19 vaccine acceptability	
9.Uzochukwu et al (2021)	Younger people and elderly respondents aged 61–80 years old were the least hesitant about the COVID-19 vaccine Roman Catholics and Protestants were more likely to accept the COVID-19 vaccine		
10. Adigwe (2021)	Older respondents were more likely to pay for COVID-19 vaccine Male respondents associated with willingness to pay for COVID-19 vaccines	Previous COVID-19 infection linked to willingness to expend resources in order to ensure protection from future exposure	
11. Eze et al (2021)	Male gender associated with COVID-19 vaccine acceptance Higher level of education associated with COVID-19 vaccine uptake Religion- Islamic religion associated with COVID-19 uptake		
12. Iliyasu et al (2022)	Higher vaccine acceptability levels were observed among males, non-Muslims, individuals with higher levels of education, high socioeconomic status and singles	Individuals concerned about COVID-19 because of their HIV-positive status associated with vaccine acceptability	Those unperturbed about the side effects of COVID-19 vaccine showed higher vaccine acceptability levels
13.Adedeji-Adenola et al (2022)	Nigerian residents aged 40–69 years likely to have a positive perception of COVID-19 vaccines	Willingness to receive COVID-19 vaccines higher among healthcare providers and those with prior exposure to COVID-19 infection	
14.Harapan et al., (2022)	Respondents in the older age group were associated with vaccine acceptability Living in the urban area, higher income earners and being Christian agnostic or Atheist were associated with vaccine acceptability	Healthcare workers less hesitant compared to those that are non-healthcare workers	Receiving flu vaccination in the past few months indicates a belief in the efficacy and benefits of vaccination also statistically significantly associated with COVID-19 vaccine acceptability
15.Anjorin et al (2021)	Older age group Urban dwellers associated with COVID-19 vaccine acceptability in this study conducted in African countries including Nigeria	Those who knew someone who got sick with COVID-19 had higher vaccine acceptance levels	Those who have taken other vaccines in the past were associated with higher levels of vaccine uptake

In addition, the cross-country comparable, descriptive high-frequency phone study showed a statistically significant association between low-income earners and low levels of education and COVID-19 vaccine uptake (Kayanda et al. 2021). This contradicts the overall picture with regard to socio-demographic predictors of the acceptability of the COVID-19 vaccine. However, the author noted in the limitation section of the study that the population-level representativeness of the results reported may be constrained since the phone survey respondents in the data were not explicitly chosen to be representative of all individuals (Kayanda et al. 2021).

A mixed picture emerged concerning the association between COVID-19 vaccine uptake and socio-demographic factors like religion and marital status (Uzochukwu et al. 2021; Harapan et al. 2022; Amuzie et al. 2021; Eze et al. 2021).

Another major theme identified as a determinant of COVID-19 vaccine uptake among adults in Nigeria is risk perception. Adults who perceived themselves as having higher risks of contracting COVID-19 infection had a significant association with vaccine acceptability (Iliyasu et al. 2021b). These groups include clinical staff/healthcare providers such as doctors, pharmacists, and nurses (Iliyasu et al. 2021b). Other high-risk groups consist of individuals with underlying health problems and chronic medical conditions such as hypertension, diabetes, and HIV/AIDs (Iliyasu et al. 2022). Lastly, concern about safety and efficacy was also identified as a critical factor influencing COVID-19 vaccination uptake among adults in Nigeria. For instance, high vaccine uptake was observed among those who were unperturbed about claims that COVID-19 vaccines are unsafe and can cause infertility (Iliyasu et al. 2021a).

#### Barriers to the uptake of COVID-19 vaccine among adults in Nigeria

The major themes identified as barriers to the uptake of the COVID-19 vaccine among adults in Nigeria include socio-demographic factors, perception of risk, concern about vaccine safety and efficacy, political factors, conspiracy theory and the cost (Table 3).

**Table 3:** Barriers to the uptake of COVID-19 vaccine among adults in Nigeria

S/N	Socio-demographic factors	Perception of risk factors	Concerns about safety and efficacy	Political factors	Conspiracy Theory	Cost
1. Iliyasu et al., (2021b)	Younger age was statistically significantly associated with vaccine hesitancy Low socioeconomic status	Individuals without underlying health issues Healthcare workers with less than 5 years of work experience Non-clinical staff at the hospital were less likely to accept COVID-19 vaccines	Concern about vaccine safety	Mistrust of authority and government policies were statistically significantly associated with vaccine hesitancy	Believe in conspiracy theories	
2. Adebisi et al (2021)	Being respondents from Northern parts of Nigeria associated with vaccine hesitancy The older age group were hesitant towards the uptake of **hypothetical** COVID-19 vaccine		Believe that clinical trials are unreliable and vaccines are not safe			
3. Iliyasu et al., (2021a)	Younger respondents Low-income earners Being respondents from Northern parts of Nigeria associated with low vaccine acceptability	Absence of Chronic medical disorder	Concerns about vaccine safety, efficacy and side effects	Mistrust for authorities Doubt about the existence of COVID-19 disease	Believe in conspiracy theories and infertility-related rumours	
4. Agha et al (2021)	Lower level of education significantly associated with COVID-19 vaccine hesitancy	Lower level of vaccine observed among nurses and midwives		Healthcare workers who felt that the National Primary healthcare development agency (NPHDA) was not managing COVID-19 well		
5. Kayanda et al. (2021)	Vaccine acceptance is lower among females Vaccine hesitancy was observed among rural dwellers Vaccine hesitancy was observed in those with more years of education		Strong safety concerns and side effects of COVID-19 vaccines associated with vaccine hesitancy	Lack of trust and dissatisfaction with the crisis management policy of the government		
6. Okafor et al (2021)	Older respondents less willing to take **hypothetical** COVID-19 vaccine Being Christians and Traditionalists statistically significantly associated with COVID-19 vaccine hesitancy				Unwillingness to be vaccinated related to misinformation on the 5G technology and Gates foundation during the early months of the pandemic	
7. Solis-Arce et al., (2021)	Women and respondents younger than 25 years less willing to take the COVID-19 vaccine		Concern about side effects and vaccine safety due to the rapid pace of vaccine developments			
8. Amuzie et al (2021)	Vaccine hesitancy is higher among young healthcare workers (aged 20–29 years), low-income earners and singles	Being a non-clinical hospital staff is significantly associated with vaccine hesitancy				
9. Uzochu kwu et al., (2021)	Married individuals, Pentecostals, Sabbatarians, and older people (26–60 years) are more hesitant than younger people					
10. Adigwe et al., (2021)			Population concern about safety and side effects associated with COVID-19 vaccine hesitancy			Paying for COVID-19 vaccination may reduce uptake
11. Eze et al (2021)	Female gender associated with vaccine hesitancy					
12. Iliyasu et al (2022)			vaccine acceptance was lower among persons who were not concerned about the potential effects of COVID-19/HIV co-infection		Believe in conspiracy theories and infertility-related rumours	
13. Adedeji- Adenola et al., (2022)	Younger age group	Non-healthcare workers associated with vaccine hesitancy	Those without prior COVID-19 infection diagnosis are more vaccine-hesitant			
14. Harapan et al., (2022)	Females, younger age groups and Muslims are more hesitant to Vaccination	Participants working in non-healthcare related job Likely to be more hesitant towards the COVID-19 vaccine	Those who had not taken a flu vaccination during the past 12 months were also more vaccine hesitant			
15. Anjorin et al (2021)	Younger participants and rural dwellers associated with vaccine hesitancy		Those who believe that their risk of getting severely sick if infected is very low and those who have refused vaccines in the past are more hesitant			

Socio-demographic factors such as low socioeconomic status, younger age group, female gender and living in rural areas are statistically significantly associated with COVID-19 vaccine hesitancy among adults in Nigeria (Iliyasu et al. 2021a; Anjorin et al. 2021; Harapan et al. 2022; Adedeji-Adenola et al. 2022; Amuzie et al. 2021; Eze et al. 2021). Conversely, surveys that were carried out online on various social media platforms in order to determine whether or not adults in Nigeria would accept a hypothetical COVID-19 vaccine produced a divergent result in terms of the socio-demographic factors that are responsible for vaccine hesitancy. The outcome of these surveys showed that the older age groups were more vaccine-hesitant (Adebisi et al. 2021; Okafor et al. 2021). Studies, however, have shown that response/social bias is possible in self-administered social media-based studies. Furthermore, because social media users are primarily young adults, older adults, people from lower socioeconomic levels, specific geographical locations, people with lower educational attainment (illiterates), and people without internet access, may inadvertently be excluded from the study (Adebisi et al. 2021). Other socio-demographic factors like religion and marital status did not show a consistent association with vaccine hesitancy.

Perception of risk also plays a critical role in COVID-19 vaccine hesitancy in Nigerian adults. Study participants working in non-healthcare-related jobs, non-clinical hospital staff and individuals without comorbidities were statistically significantly associated with vaccine hesitancy (Agha et al. 2021; Harapan et al. 2022; Adedeji-Adenola et al. 2022; Amuzie et al. 2021; Iliyasu et al. 2021b).

Concern about vaccine safety and efficacy as a theme also emerged as a major barrier to the uptake of the COVID-19 vaccine. Individuals who believe clinical trials are unreliable, those concerned about side effects and vaccine safety due to rapid vaccine development have all demonstrated vaccine hesitancy (Solís Arce et al. 2021; Anjorin et al. 2021; Harapan et al. 2022; Adigwe 2021; Adedeji-Adenola et al. 2022; Kayanda et al. 2021; Iliyasu et al. 2021b).

Conspiracy theories surrounding the COVID-19 pandemic such as misinformation that the pandemic is related to the introduction of 5G technology and the possibility of COVID-19 vaccines causing infertility also significantly associated with vaccine hesitancy among adults in Nigeria (Iliyasu et al. 2021a; Okafor et al. 2021). In addition, requesting payment for the COVID-19 vaccine from the public has also been linked to lower vaccination rates (Adigwe 2021).

## Discussion

As already established, vaccine hesitancy predates the COVID-19 pandemic and poses a threat to world health because it is implicated in the resurgence of vaccine-preventable diseases such as measles and poliomyelitis. Similarly, the global attempt to contain the present epidemic with its detrimental health and socioeconomic repercussions may be limited by COVID-19 vaccine hesitancy (Sallam et al. 2021). Current estimates based on global data indicate that only 13.0% of Nigerians have received a full dosage of COVID-19 vaccinations, and this is significantly less than the 60.0–75.0% required to acquire herd immunity in any community (Ritchie et al. 2022). It is therefore critical to identify the factors responsible for COVID-19 vaccine hesitancy among various subgroups of the Nigerian population to aid policymakers in the establishment of effective interventions to address these issues.

This study shows huge variability in COVID-19 vaccine acceptance rates among various subgroups of the Nigerian population (24.3%–86.2%). This is consistent with other studies conducted on COVID-19 vaccine acceptance in different parts of the world. For instance, substantial heterogeneity was reported in the acceptance rates of the COVID-19 vaccine across the globe with acceptance rates ranging from 23.6% to 97.0% (Sallam et al. 2021). Despite the variability of acceptance rates observed in this study, certain patterns, however, can be deduced from the descriptive analysis of the reported vaccine acceptance rates.

The acceptance rates of the COVID-19 vaccine among high-risk populations range from 24.3 to 49.5%. These rates are not as high as expected considering the fact that such individuals are aware of the possibility of having severe forms of COVID-19 infection due to the presence of underlying health issues and higher levels of exposure.

However, the outcome is consistent with findings from previous studies conducted among healthcare providers around the world. For instance, an acceptance rate of 40.0% was reported among nurses in Hong Kong, while an acceptance rate of 36.0% was noted among healthcare workers in New Mexico (Wang et al. 2020; Shekhar et al. 2021). On the other hand, the acceptance rates observed among low-risk adults in Nigeria range from 26.0 to 86.2%. These results corroborate findings from previous studies conducted in other parts of the world. For instance, acceptance rates of 65.0% and 69.0% were reported among adults in Ireland and the United Kingdom, respectively, while the outcome of a similar study among working adults in France revealed a vaccine acceptance rate of 71.2% (Murphy et al. 2021; Schwarzinger et al. 2021).

Findings from this study show that acceptance rates of the COVID-19 vaccine among the low-risk adult population were higher compared to the high-risk subgroup in Nigeria. This is similar to studies conducted in the USA where an acceptance rate of 36.0% was reported among healthcare providers while a higher acceptance rate of 67.0% was recorded in a general population survey conducted in the USA (Malik et al. 2020). It is therefore imperative to evaluate factors that are predictive of vaccine acceptance among high-risk populations, such as healthcare workers, as this will assist organisations and policy-makers in allocating resources in a manner that will optimise vaccine uptake in the subgroup.

Limited access to scientific information about COVID-19 vaccine research and the approval process have been identified as factors contributing to COVID-19 vaccine hesitancy, particularly among high-risk groups such as healthcare providers (Garrett 2020). In a study of US healthcare workers, 56.0% preferred to wait and evaluate more data on the COVID-19 vaccine before accepting it. This is not surprising given that healthcare workers prefer to base their decisions on published scientific literature rather than anecdotal evidence (Shekhar et al. 2021). Also, the acceptability of COVID-19 vaccine among healthcare workers in Nigeria was assessed using the Fogg Behaviour Model in July 2021, after the arrival of the vaccine, and it was reported that only 33.0% of the study participants had taken two doses of the vaccine (Agha et al. 2021). The study further clarified the reasons for the unexpected low vaccine uptake among high-risk populations like healthcare workers, as it was observed that low motivation and low ability were powerful predictors of COVID-19 vaccine hesitancy. In addition, though high acceptability was reported in most surveys conducted among the low-risk subgroup of the adult populations in Nigeria, some of these surveys assessed the willingness to accept a hypothetical COVID-19 vaccine. Some researchers, however, argued that willingness to accept a vaccine does not always translate to vaccine uptake eventually (Adebisi et al. 2021).

In terms of factors responsible for the uptake of the COVID-19 vaccine, studies conducted in the USA showed that vaccine acceptance increased with increasing age, education, male gender, and income level (Shekhar et al. 2021; Malik et al. 2020). The outcome of this review is consistent with such a claim because the older age group, male gender, higher level of education, and high socioeconomic status were repeatedly mentioned in the reviewed studies as predictors of acceptability of the COVID-19 vaccine (Iliyasu et al. 2021a; Uzochukwu et al. 2021; IMF 2020; Agha et al. 2021; Eze et al. 2021).

Other factors acting as facilitators of COVID-19 vaccine uptake in this study include high-risk perception; for instance, individuals who are frontline healthcare providers and those with chronic medical conditions were found to be less vaccine-hesitant in some studies. Also, receiving flu vaccination in the past few months, which indicates a belief in the efficacy and benefits of vaccination, was found to be statistically significantly associated with COVID-19 vaccine uptake (Anjorin et al. 2021; Harapan et al 2022). These results are consistent with studies conducted in Northern Italy where past vaccination refusal and absence of comorbidities were reported as important predictors of COVID-19 vaccine refusal (Reno et al. 2021).

The barriers revealed in this study include socio-demographic factors, perception of risk, concern about vaccine safety and efficacy, political factors, conspiracy theories, and cost. This review, therefore, has demonstrated that COVID-19 vaccine hesitancy in Nigeria is due to a complex interplay of many factors at different levels. Socio-demographic variables such as younger age group, low socio-economic status, female gender, and lower level of education were revealed as barriers to the uptake of the COVID-19 vaccine. Also, individuals who perceive themselves as having a low risk of contracting COVID-19 infection were found to be vaccine-hesitant in this study. This corroborates other studies conducted in other parts of the world. For instance, it was discovered that outright vaccine refusal and vaccine reluctance among working adults in France were both substantially associated with female gender, age, lower educational level, poor compliance with recommended immunisations in the past, and no report of defined chronic diseases (Schwarzinger et al. 2021). Invincibility, low-risk perception, and the rebellious character of young adults are all possible explanations for vaccine hesitancy among young adults in Nigeria (Iliyasu et al. 2021a).

With regard to concerns about vaccine safety, studies have shown that beliefs that vaccinations aren't safe or effective, as well as growing anxiety over the quick development of COVID-19 vaccines, are all contributing to vaccine hesitancy (Aw et al. 2021). Similarly, the findings of this study established vaccine safety and efficacy concerns as a substantial barrier to COVID-19 vaccine uptake. People's perceptions of COVID-19 vaccines in Nigeria include the ideas that they are unsafe and that clinical trials are not trustworthy (Solís Arce et al. 2021; Adigwe 2021; Adebisi et al. 2021; Kayanda et al. 2021; Iliyasu et al. 2021b).

Furthermore, this review also established that COVID-19 vaccine hesitancy is strongly associated with a negative relationship between the government and the citizens. Distrust in the government and dissatisfaction with the COVID-19 crisis management policies of the government are important predictors of COVID-19 vaccine hesitancy (Iliyasu et al. 2021a). This result corroborates other studies’ findings on determinants of COVID-19 vaccine hesitancy. For instance, in a study conducted among eligible Japanese adults, mistrust towards the government was mentioned as one of the reasons for the refusal of the COVID-19 vaccine (Okubo et al. 2021). Similarly, studies have shown that having a positive view of public sector officials and the UK government led to a significant increase in willingness to get vaccinated (Chaudhuri et al. 2022). In addition, it was discovered that people in Australia were more likely to plan to get vaccinated if they had more confidence in their state or territory government or hospitals (Edwards et al.^2021^).

The outcome of various studies conducted all over the world showed that the existence of a great number of conspiracy theories and false narratives may act as substantial hurdles to the uptake of COVID-19 vaccinations. The findings of this study support this assertion, as the inhibitory effects of conspiracy theories on vaccine uptake have been observed in some of the studies reviewed (Iliyasu et al. 2021a). Finally, cost is a critical determinant of COVID-19 vaccine hesitancy among Nigerian adults because only 26% of respondents were willing to pay to receive the COVID-19 vaccine among respondents (Adigwe 2021).

In a rapid review study of the COVID-19 vaccine acceptance rate and the associated factors in Nigeria conducted in December 2021, it was reported that propaganda, conspiracy theories, and worries about harmful effects were the root causes of COVID-19 vaccine hesitancy (Olu-Abiodun et al. 2022). This systematic review has, however, provided fresh insights on anti-COVID-19 vaccination behaviour in various subgroups of adults in Nigeria and the mechanisms that underpin it, by uncovering additional factors such as cost, perception of risk, political and socio-demographic factors as major barriers to the uptake of the vaccine in Nigeria. On the whole, there is a need for policies to address these issues for the Nigerian government to be able to achieve its goal of vaccinating 70% of 200 million people by 2022 (Usigbe 2021).

Nigeria got 3.92 million doses of COVID-19 vaccination through the COVAX facility on March 2, 2021. The arrival represented a significant milestone toward assuring equitable global distribution of COVID-19 vaccinations in the largest vaccine procurement and supply operation in history (WHO 2021). However, due to limited supply, the vaccine was rolled out in stages to different subgroups of the population, with healthcare workers being one of the first subgroups to receive it in Nigeria, as was the case in the United States. For the first phase of the COVID-19 vaccine roll-out, which mainly targeted frontline workers, 98.9% (3,980,600 doses) of the first tranche of Astra Zeneca vaccines were used in the first phase, with over 2.5 million people having received the 1st dose of the vaccines, of which over 1.4 million people have received the 2nd dose, reaching 2.3% of the eligible population (WHO 2021).

By the time the second phase of COVID-19 vaccination was launched in August 2021, the reasons for poor vaccine coverage had shifted from inadequate vaccine supply to low turnout and suboptimal COVID-19 vaccine uptake caused by the factors enumerated above.

### Strengths and limitations

One of the main strengths of this study is the screening procedure adopted for the included citations. Each citation was screened by two reviewers, and disagreements were resolved by a senior author. The same was done for quality assessment to ensure robust evidence. This study also demonstrated the usefulness of systematic reviews in revealing knowledge gaps associated with a given issue.

On the other hand, the following are some of the study's limitations. Some of the studies reviewed used survey data, which is susceptible to selection, response, and recall biases. During the pandemic, many researchers resorted to conducting COVID-19 disease-related studies online because leveraging the internet for studies during the outbreak minimised contact and the associated COVID-19 infection risk while generating valuable insights from these studies. So, many of the surveys conducted among low-risk populations were either internet, telephone, or social media-based studies. However, conducting studies online may inadvertently result in the selection of a more urban, young-to-middle-aged, and highly educated population while excluding older adults, people from lower socioeconomic levels, particular geographical places, lower educational attainment, and people without internet access (Adebisi et al. 2021). This may subsequently limit the generalisability of the studies reviewed since the online participants may not truly represent the population in a country like Nigeria. Also, measurement errors may occur in studies conducted with population-based data, leading to information bias.

## Conclusions

Substantial variability in COVID-19 vaccine acceptance rates was observed among different subgroups of adults in the Nigerian population. More than half of the studies reviewed reported acceptance rates below 60.0%, and this could negatively impact efforts to control the current COVID-19 pandemic. Factors impacting the acceptance and refusal of the COVID-19 vaccine in Nigeria include socio-demographic factors; perception of risk; concern about vaccine safety and efficacy; political factors; conspiracy theory; and cost.

Furthermore, high motivation and ability have been identified as important predictors of COVID-19 vaccine uptake among healthcare workers (Agha et al. 2021). It is therefore imperative to further research into factors responsible for low motivation and low ability among healthcare workers and mitigate them to improve COVID-19 vaccine uptake among the subgroup. It is crucial to increase vaccination rates among healthcare workers and provide them with evidence-based information regarding the COVID-19 vaccine for the following reasons. Firstly, increased COVID-19 vaccine uptake is essential to curtail the spread of the infection among healthcare providers and prevent the loss of critical manpower needed to combat the pandemic. In addition, healthcare providers are a primary source of health education and literacy in the community; thus, they must be better prepared to counter arguments against COVID-19 vaccination uptake and challenge mistrust at all levels of the community.

In general, government agencies, policymakers, public health institutions, non-governmental organizations (NGOs), advocacy groups, mainstream media, and social media operators must collaborate to overcome the challenge of COVID-19 vaccine hesitancy. Because they influence the public, the Nigerian government should also work towards gaining the trust of key stakeholders and opinion leaders such as market heads, religious leaders, celebrities, traditional and cultural leaders so they can assist government agencies in tackling COVID-19 vaccine hesitancy by effectively engaging members of the community.

When health promotion activities are supported by health promotion theories such as the diffusion of innovations model, nudge theory, trans-theory model, and health belief model, they are more successful (Eldredge et al. 2016). Relevant government agencies should therefore create targeted community-based interventions based on these theories.

Disseminating scientific and evidence-based information on how safe and effective licensed COVID-19 vaccines are through digital as well as traditional media such as radio, television, and print would also help ensure that accurate information reaches remote regions of the country because everyone needs evidence-based information. This will boost the confidence of Nigerian citizens in the COVID-19 vaccine and increase their willingness to accept it (Olu-Abiodun et al. 2022).

## Data Availability

Data supporting this systematic review are available in the reference section. In addition, the datasets used or analysed during the current study are available from the corresponding author on reasonable request.

## Abbreviations

CDC: Centre for disease control
COVID-19: Coronavirus disease 2019
COVAX: COVID-19 vaccines global access
HIV: Human immunodeficiency virus
HPV: Human papilloma virus
IMF: International monetary fund
NCDC: Nigerian centre for disease control
PRISMA: Preferred reporting items for systematic reviews and meta-analyses
SWiM: Synthesis without meta-analysis
UK: United Kingdom
USA: United States of America
WHO: World health organisation
